# Proliferation PET/CT Imaging of Salivary Gland Tumor

**DOI:** 10.3390/diagnostics11112065

**Published:** 2021-11-08

**Authors:** Ryogo Minamimoto

**Affiliations:** National Center for Global Health and Medicine, Department of Radiology, Division of Nuclear Medicine, Tokyo 162-8655, Japan; ryogominamimoto@yahoo.co.jp; Tel.: +81-3-3202-7181; Fax: +81-3-3207-1038

**Keywords:** 4DST PET/CT, cell proliferation imaging, FDG PET/CT, salivary gland tumor, pleomorphic adenoma, Warthin’s tumor

## Abstract

Salivary gland tumors are rare neoplasms which vary in terms of origin and malignant potential. 2-[18F]-fluoro-2-deoxy-d-glucose (FDG)-positron emission tomography (PET) has limited ability to differentiate between different types of salivary gland tumors because both Warthin’s tumors and pleomorphic adenomas usually show increased FDG uptake, with no statistically significant difference in standardized uptake value (SUV) compared with malignant salivary gland tumors. Here, we discuss 4′-[methyl-11C]-thiothymidine (4DST) PET, which provides cell proliferation imaging capable of demonstrating intense uptake in parotid carcinoma and Warthin’s tumor, but no uptake in parotid pleomorphic adenoma. This is the first report of the potential of proliferation PET/ computed tomography (CT) imaging for characterizing salivary gland tumors based on the molecular pathogenesis of the tumor.

4DST is a PET tracer for cell proliferation imaging that is incorporated into DNA [1]. Previous studies have confirmed a higher correlation with proliferation of lung tumors and renal cell cancer for 4DST than for FDG [2,3]. The potential of 4DST PET/CT for characterizing head and neck squamous cell carcinoma has been reported, with uptake values lower than those for FDG, which shows a similar tendency to the present case [4] (Figure 1).

FDG-PET/CT generally shows intense uptake in Warthin’s tumors, mimicking malignancy. The numerous mitochondria, immunoglobulin A, and lymphoid stroma in the epithelial cells of Warthin’s tumors cause increased glucose metabolism that leads to increased FDG uptake in the tumors [5].

[18F]-fluoro-3′-deoxy-3′-L-fluorothymidine (FLT) uptake, representing cell proliferation related to the salvage pathway of DNA synthesis had faint uptake (SUV_max_ 1.9), whereas there was positive FDG uptake (SUV_max_ 3.2) in Warthin’s tumor, in which no Ki-67-positive cells were found [6]. Faur et al., assessed proliferative activity in Warthin’s tumors based on Ki-67 expression, and reported that most tumors showed low Ki-67 (<5%) [7]. Flow cytometric analysis of the DNA content in Warthin’s tumors showed a low S-phase fraction + G2- plus M-phase fraction with low Ki-67. DNA aneuploidy was not detected in the Warthin’s tumors, indicating their low proliferative activity [8]. Kuzenko et al., found a balanced distribution of epithelial and stromal components in Warthin’s tumors and positive Ki-67 staining in basal cells of the epithelium and lymphoid tissue [9]. Accordingly, the basal cells of the epithelium and the abundant lymphoid tissue of Warthin’s tumors are possibly related to uptake in proliferation PET; however, given their slow-growing nature, 4DST uptake might be affected by reactive lymphoid tissue rather than the epithelium. Similarly, 4DST uptake was confirmed in cardiac sarcoidosis, characterized as granuloma formation with the presence of replicating lymphocytes, which correlated with disease activity [10]. The present case found that proliferation PET can characterize Warthin’s tumors similarly to FDG-PET/CT, but the uptake mechanism appears to differ between the two techniques (Figure 2).

Pleomorphic adenoma (PA) is one of the most common benign neoplasms of the salivary glands and is characterized as an encapsulating tumor with epithelial and myoepithelial components in addition to mesenchymal and stromal components. High GLUT-1 immunoexpression (>25% immunostained cells) has been reported in 57% of PA [11], and the expression of GLUT-1 was found to significantly correlate with FDG uptake [5]. Low Ki-67 index has been reported for PA, in the range 0.8–6.7% [12,13,14,15,16,17,18,19]. In carcinoma ex-pleomorphic adenoma (CXPA), defined as a carcinoma arising from a primary or recurrent benign PA, Ki-67 index was high (49.3%). However, that in residual areas of PA in CXPA (6.9%) was almost equivalent to that of PA (6.7%) (16). The characteristic 4DST PET finding for PA is low proliferation, which can contribute to differentiation of PA from malignant tumors (Figure 3).

In conclusion, we report the potential of proliferation PET/CT imaging for characterizing salivary gland tumors based on the molecular pathogenesis of the tumor. Further study is required to clarify the utility of 4DST-PET/CT for the assessment of salivary gland tumors.

## Figures and Tables

**Figure 1:**
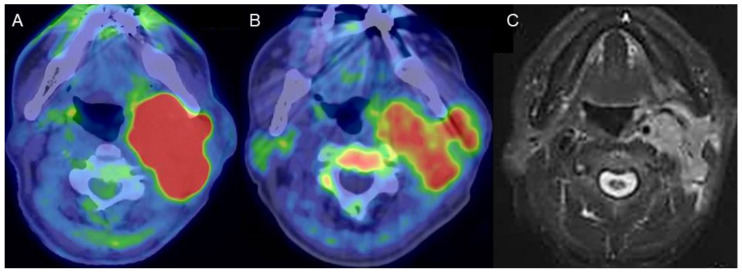
A man in his 80s with carcinoma arising in the left parotid gland. (**A**) Fused FDG-PET/CT image (axial view), (**B**) fused 4DST PET/CT image (axial view), and (**C**) gadolinium (Gd)-enhanced MRI (axial view). Gd-enhanced MRI shows homogeneous enhancement of an irregular-shaped lesion in the left parotid gland. High FDG uptake (SUV_max_: 21.8) as well as lower, but unequivocal, 4DST uptake (SUV_max_: 6.0) were confirmed in the lesion.

**Figure 2:**
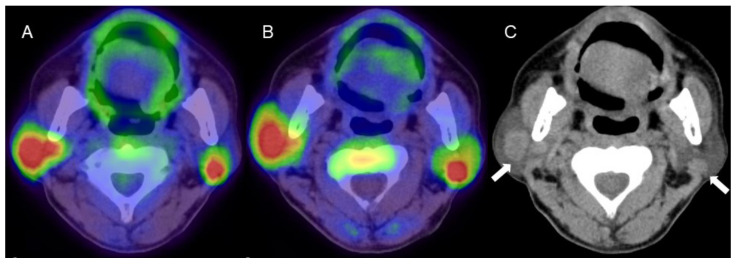
A man in his 60s with pathologically proven Warthin’s tumor in the parotid glands bilaterally. (**A**) Fused FDG-PET/CT image (axial view), (**B**) fused 4DST PET/CT image (axial view), and (**C**) plain CT (axial view). Intense uptake of both FDG (SUV_max_: 9.6) and 4DST PET/CT (SUV_max_: 12.0) is seen in bilateral nodular lesions, which show high intensity on plain CT (arrows) consistent with the typical features of Warthin’s tumors. Aspiration biopsy revealed oncocytes and abundant lymph-node cells and epithelium with eosinophilic cytoplasm, and a diagnosis of Warthin’s tumor was made based on pathological findings.

**Figure 3:**
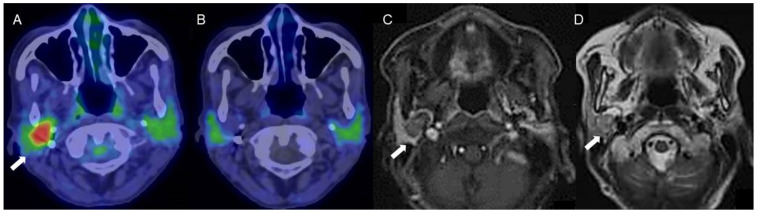
A woman in her 70s with a tumor of the right parotid gland, highly suspected to be pleomorphic adenoma (arrows). (**A**) Fused FDG-PET/CT image (axial view), (**B**) fused 4DST PET/CT image (axial view), (**C**) Gd-enhanced MRI (axial view), and (**D**) T2WI MRI image (axial view). On T2WI, a well-circumscribed intraparotid mass of intermediate-to-high signal and a low-signal rim is seen, with heterogeneous nodular enhancement, characteristic of pleomorphic adenoma [20]. The tumor shows increased FDG uptake (SUV_max_: 5.2) but no 4DST uptake.

## Data Availability

Not applicable.

## Abbreviations

CXPA:	carcinoma ex-pleomorphic adenoma
FDG:	2-[18F]-fluoro-2-deoxy-d-glucose
FLT:	[18F]-fluoro-3′-deoxy-3′-L-fluorothymidine
Gd:	gadolinium
PA:	Pleomorphic adenoma
PET:	positron emission tomography
SUV:	standardized uptake value
4DST:	4′-[methyl-11C]-thiothymidine
